# Characterization of miRNAs in Extracellular Vesicles Released From Atlantic Salmon Monocyte-Like and Macrophage-Like Cells

**DOI:** 10.3389/fimmu.2020.587931

**Published:** 2020-11-11

**Authors:** Nicole C. Smith, Gabriel Wajnberg, Simi Chacko, Nardos T. Woldemariam, Jacynthe Lacroix, Nicolas Crapoulet, D. Craig Ayre, Stephen M. Lewis, Matthew L. Rise, Rune Andreassen, Sherri L. Christian

**Affiliations:** ^1^ Department of Ocean Sciences, Memorial University, St. John’s, NL, Canada; ^2^ Atlantic Cancer Research Institute, Moncton, NB, Canada; ^3^ Department of Life Sciences and Health, OsloMet-Oslo Metropolitan University, Oslo, Norway; ^4^ Department of Molecular Sciences, University of Medicine and Health Sciences, Basseterre, Saint Kitts and Nevis; ^5^ Department of Chemistry & Biochemistry, Université de Moncton, Moncton, NB, Canada; ^6^ Beatrice Hunter Cancer Research Institute, Halifax, NS, Canada; ^7^ Department of Biochemistry, Memorial University, St. John’s, NL, Canada

**Keywords:** extracellular vesicles, microRNA, Atlantic salmon, RNA-sequencing, RNA-seq, macrophage, head kidney culture

## Abstract

Cell-derived extracellular vesicles (EVs) participate in cell-cell communication *via* transfer of molecular cargo including genetic material like miRNAs. In mammals, it has previously been established that EV-mediated transfer of miRNAs can alter the development or function of immune cells, such as macrophages. Our previous research revealed that Atlantic salmon head kidney leukocytes (HKLs) change their morphology, phagocytic ability and miRNA profile from primarily “monocyte-like” at Day 1 to primarily “macrophage-like” at Day 5 of culture. Therefore, we aimed to characterize the miRNA cargo packaged in EVs released from these two cell populations. We successfully isolated EVs from Atlantic salmon HKL culture supernatants using the established Vn96 peptide-based pull-down. Isolation was validated using transmission electron microscopy, nanoparticle tracking analysis, and Western blotting. RNA-sequencing identified 19 differentially enriched (DE) miRNAs packaged in Day 1 versus Day 5 EVs. Several of the highly abundant miRNAs, including those that were DE (e.g. ssa-miR-146a, ssa-miR-155 and ssa-miR-731), were previously identified as DE in HKLs and are associated with macrophage differentiation and immune response in other species. Interestingly, the abundance relative of the miRNAs in EVs, including the most abundant miRNA (ssa-miR-125b), was different than the miRNA abundance in HKLs, indicating selective packaging of miRNAs in EVs. Further study of the miRNA cargo in EVs derived from fish immune cells will be an important next step in identifying EV biomarkers useful for evaluating immune cell function, fish health, or response to disease.

## Introduction

Extracellular vesicles (EVs) are cell-derived, lipid bilayer-enclosed particles that are secreted from many, if not all, cell types, including immune cells (1–3). Three categories of EVs have been described: exosomes (30–100 nm in diameter), which are formed when multivesicular bodies fuse with the plasma membrane to release intraluminal vesicles; microvesicles (100–1000 nm in diameter), which are formed from direct budding of the plasma membrane; and apoptotic bodies (>1 μM in diameter), which are formed from the blebbing membrane of an apoptotic cell (4, 5). For the purpose of this study, the term EV will refer to exosomes and microvesicles since, due to our isolation methods, large apoptotic bodies are unlikely to represent a major contribution to the observed results. EVs share some common characteristics, which enable their identification from cells and other particles. Among the most robustly validated canonical markers are membrane-associated proteins such as heat shock proteins (HSP70, HSP90) and certain members of the tetraspanin superfamily of proteins (CD9, CD63, CD37, CD81, CD82) (4, 6). While EVs have been widely studied in mammals, there are only a few studies that examine EVs in teleost fish, which will be discussed below (7–12).

EVs participate in cell-cell communication *via* transfer of their molecular cargo, which can include messenger RNA (mRNA), microRNA (miRNA), DNA, and protein (2, 13). In mammals, EVs have been implicated in many physiological and pathological processes, including immune cell regulation and host-pathogen interactions (14, 15). Phagocytic immune cells have been shown to use EVs as a mechanism to regulate neighbouring cells within their environment. For example, pathogen-challenged macrophages release EVs containing pathogen associated molecular patterns (PAMPs) that stimulate recipient cells to produce cytokines including IL-10, IFNγ, TNFα, and IL-1β (16–19). Similarly, B cells and dendritic cells can use EVs carrying surface-bound MHC I and II molecules to present antigens and stimulate T cell activation (14, 20, 21). EVs are derived from cells under both normal and pathological conditions, and their molecular cargo is reflective of their cell of origin. For instance, tumour cells have been shown to release EVs containing tumour-specific miRNAs (22). Therefore, EVs can also serve as biomarkers for health and disease (23).

Mature miRNAs are short (~22 nucleotides), non-coding RNAs that play a key role in the regulation of biological processes *via* post-transcriptional regulation of gene expression (24–26). As part of the RNA induced silencing complex (RISC) the mature guide miRNAs downregulate gene expression by binding to partially complementary mRNA sequences to either block their translation or induce their degradation (25). EVs can transfer miRNAs between cells where they can regulate the expression of various genes, including those relevant for cell differentiation and immune response (27–29). In teleost fish, miRNAs have been reported to be involved in cell differentiation, growth, reproduction, and regulation of immune responses (30, 31). For example, miR-21 modulates the inflammatory response in miiuy croacker (*Miichthys miiuy)* and grass carp (*Ctenopharyngodon idella*) following *Vibrio anguillarum* and *Aeromonas hydrophila* infection, respectively, and miR-155 is associated with the immune response of several fish species following viral challenge (31–33). Additionally, small RNA deep sequencing has identified differential miRNA expression in multiple tissues of various teleost fish species following pathogen exposure (30, 31, 34–36). Some miRNAs involved in the teleost immune response are also associated with the immune response of mammals, suggesting the function of these miRNAs may be evolutionarily conserved (31, 37). However, putative fish-specific miRNAs, such as miR-2188 and miR-731, play a role in the immune response of several fish species, but have not been described in mammals (34, 38–41).

Macrophages play a critical role in initiating an immune response through several processes including phagocytosis, production of reactive intermediates, and production of cytokines and other pro- and anti-inflammatory proteins (42, 43). Two major types of macrophages have been characterized depending on their activation and cytokines produced: M1 (pro-inflammatory) and M2 (anti-inflammatory) (42, 43). While M1 macrophages are involved in the ability to respond to pathogenic challenge, M2 macrophages are involved in processes such as tissue remodeling, fibrosis and wound repair (42, 43). In fish, an adherent population of leukocytes, consisting of multiple cell types including macrophages and their precursors monocytes, can be isolated and cultured from the anterior (or head) kidney, which is the main site of hematopoiesis in fish and equivalent to the mammalian bone marrow (44–46). Based on morphology, phagocytic ability, and miRNA profile, our previous research suggested that Atlantic salmon head kidney leukocytes (HKLs) change *in vitro* from primarily monocyte-like at Day 1 of culture to primarily macrophage-like at Day 5 of culture (47). Therefore, we analyzed the miRNA profile of EVs released from these two cell populations. If differentially packaged miRNAs are present in the two populations, they may help distinguish EVs released by monocytes or progenitor cells (Day 1) from EVs released from macrophages (Day 5). This is particularly relevant for health and disease monitoring. Monocytes represent a comparatively naïve, steady-state cell type, whereas their differentiation into macrophages is associated with active immunity, response to pathogenic conditions, and antigen presentation (48–50). As such, identifying EVs with differences in miRNA abundance between monocytes and macrophages could provide a means for quantifying the activity of the immune system.

Studying EVs and their packaged cargo in teleost fish may aid in the identification of biomarkers of health, disease and/or response to environmental stressors. Using Q-TOF mass spectrometry (MS), proteins including MHCIIB, HSP70 and HSP90 were identified in EVs derived from Atlantic salmon leukocytes that were stimulated with cytosine–phosphate–guanosine (CpG) oligonucleotides, an established PAMP analogue (7, 12). Similarly, proteins including granulins, MHCI, MHCII, and proteasome subunits were identified in serum-derived EVs from Atlantic salmon infected with *Piscirickettsia salmonis* (8). In rainbow trout (*Oncorhynchus mykiss*), it was demonstrated that heat shock induced the release of HSP70 enriched exosomes *in vivo* isolated from plasma, and *in vitro* isolated from cultured hepatocytes (10). The differential loading of EV cargo molecules, including miRNAs between physiological states, has been established in mammals (22, 23). If EVs have similar characteristic differences in miRNA profiles in teleost fish they may serve as molecular signatures for fish physiological state. For example, Atlantic cod (*Gadus morhua*) reared in elevated water temperature were found to have serum EVs with different protein and miRNA cargo than control Atlantic cod reared in optimal water temperature (9). Additionally, signature miRNAs corresponding to sex differentiation were identified in serum EVs of tongue sole (*Cynoglossus semilaevis)*, allowing early detection of sex differentiation, which may enhance the efficiency of reproduction and cultivation (11). Studying the miRNA cargo of fish EVs is, therefore, of considerable interest in understanding how they may be related to fish culture, health, and response to disease. The use of EVs from blood samples, for example, as opposed to more invasive biopsies, or sacrificed animals, may be used for responsive, potentially non-lethal, and timely monitoring of health in both wild and farmed fish. Characterizing EVs and their cargo derived from immune cells is a key first step in determining immune-related EV specific biomarkers.

## Materials and Methods

### Animals

The Atlantic salmon (1.5 kg +/- 0.3 kg) used for this experiment were reared in the Dr. Joe Brown Aquatic Research Building (JBARB) of the Ocean Sciences Centre and kept at 12°C with 95%–110% oxygen saturation, using a flow-through seawater system. All procedures in this experiment were approved by Memorial University of Newfoundland’s Institutional Animal Care Committee (18-01-MR; 14-02-MR), following the guidelines from the Canadian Council on Animal Care. Due to the limiting number of HKLs isolated per fish, and the low amount of RNA available in EVs, a total of 16 Atlantic salmon were used in this study: five individuals for RNA-seq (one individual was excluded from RNA-seq due to low RNA yield), five individuals for reverse transcriptase (RT)-qPCR and nanoparticle tracking analysis (NTA), three individuals for Western blot, and three individuals for transmission electron microscopy (TEM).

### Head Kidney Leukocyte Isolation

HKLs have been used in several fish immunology studies [e.g. (44, 51–53)]. In this study, HKLs were isolated as previously described in Smith et al. (47). Briefly, the HK was removed and placed in isolation media: Leibovitz-15 medium (L-15 Gibco, Carlsbad, CA, USA) supplemented with 2.5% fetal bovine serum (FBS, Gibco), 1% penicillin/streptomycin (Gibco) and 27.5 mg of heparin (Sigma-Aldrich, St. Louis, MO, USA). The HK was forced through a 100 µm nylon cell strainer (Thermo-Fisher Scientific, Waltham, MA, USA) to generate a single-cell suspension, which was then loaded onto a 34/51% Percoll (GE Healthcare, Uppsala, Sweden) gradient (prepared with UltraPure DNase/RNase-Free Distilled Water (Thermo-Fisher Scientific) and 10X Hank’s Balanced Salt Solution (HBSS; Sigma-Aldrich) and centrifuged at 500 x g for 30 min at 4˚C. Following centrifugation, the interface between the 34% and 51% gradient, which consists of leukocytes, was collected and washed twice in isolation media at 500 x g for 5 min at 4°C. The cells were re-suspended in culture media (L-15 supplemented with 5% FBS and 1% penicillin/streptomycin; held on ice), and viable cells were counted on a haemocytometer using Trypan Blue (Sigma-Aldrich) dead-cell exclusion. The cells were then seeded in 6-well culture plates (Corning, Corning, NY, USA) at 3 x 10^7^ cells in 2 mL of culture media per well and incubated at 15˚C. Six hours after plating, the cells were washed twice in culture media, leaving only the adhered cells. The media of the cells to be sampled on Day 1 was replaced with vesicle-free culture media, while the media of the remaining cells was replaced with regular culture media. Vesicle-free culture media was made as follows: culture media was prepared as described above, except with double the amount of FBS (10% FBS). The media was centrifuged at 100,000 x g for 16 h at 4°C. The supernatant was sterilized through a 0.22 μm filter and then diluted with depleted culture media (media without FBS) to reach a final concentration of 5% FBS (54). Twenty-four hours later, the media from Day 1 cells was collected and centrifuged at 1800 x g for 5 min at 4°C, followed by 17,000 x g for 15 min at 4°C, to eliminate cells and debris. The media was stored at -80°C until further processing. This procedure was then repeated for Day 5 cells where the media was replaced with vesicle-free media 24 h before sampling. In the current study’s Day 5 cultures, macrophage-like cell morphology as see in (47) was confirmed by eye. In addition, viability was assessed by lack of cell debris for each experiment.

### Transmission Electron Microscopy

The morphology of HKL-derived EVs was analyzed using TEM. Five microlitres of culture media containing EVs were placed on a copper formvar/carbon grid (Electron Microscopy Sciences, Hatfield, PA, USA) and stained with 2% Uranyl Acetate for 1 min, followed by a 1 min wash in 0.1 µM filtered phosphate buffered saline (PBS; Thermo-Fisher Scientific) at room temperature. Imaging was performed using a Tecnai Spirit Transmission Electron Microscope, equipped with a 4 megapixel AMG digital camera.

### Nanoparticle Tracking Analysis

Culture media containing EVs released from Day 1 HKLs and culture media containing EVs released from Day 5 HKLs was diluted 1:10 in 0.1 μM filtered PBS, and the concentration and size of the EVs were analyzed using a NanoSight NS300 (Malvern Panalytical, St-Laurent, Quebec, CA). Samples were applied to the NanoSight using a continuous syringe pump. The number of particles in the window was kept at 40–100 per frame. The screen gain was set to 3.0 and the camera level to 13. Five videos were recorded per sample at 60 s per video.

### Extracellular Vesicle Isolation

EVs were isolated using the Vn96 peptide (New England Peptide, Gardner, MA, USA) following the manufacturer’s instructions. Vn96 binds to at least 5 unique HSPs secreted by a variety of different cell types (55, 56). In addition, Vn96 isolates EVs with reduced contamination from protein aggregates or lipoproteins, compared to other methods of EV isolation (i.e. ultracentrifugation) (57) . Briefly, 1 ml of EV-containing media was incubated with 40 μl (2.5 mg/ml) of Vn96 for 1 h, rotating, at room temperature. Following the 1 h incubation, the EV-containing media was centrifuged at 17,000 g for 15 min at 4°C. The pellet was washed 3 times in 0.1 μm filtered PBS at 15,000 x g for 10 min at 4°C and resuspended in the appropriate buffer: 100 μl of mirVana lysis buffer for RNA isolation or 30 μl of radioimmunoprecipitation assay buffer (RIPA: 50 nM Tris-HCl, 0.02% sodium azide, 0.5% sodium deoxycholate, 0.1% SDS, 1% NP-40, 150 mM NaCl) for Western blot.

### Western Blot

All samples (Atlantic salmon head kidney and liver, Vn96 isolated EVs and murine Wehi-231 B-cells) were lysed in RIPA buffer supplemented with 1:100 of 10 mg/ml PMSF (Sigma), 1 μM aprotinin (Sigma) and 1X HALT protease inhibitor cocktail (Thermo-Fisher). Protein content of the head kidney and liver samples was determined using the bicinchoninic acid (BCA; Thermo-Fisher Scientific) assay following the manufacturer’s protocol. One, 5 and 10 μg of head kidney and liver lysate and all of the Wehi-231 lysate (from 5.0 x 10^5^ cells) or 10 μl of EV lysate were run on 10% SDS-PAGE gels followed by transfer to nitrocellulose membranes. Blocking was performed using 5% (w/v) skim milk in tris buffered saline plus tween (TBST) for 1 h at room temperature. Anti-mouse HCS 70 (B-6) (sc-7298; Santa Cruz Biotechnology Inc., Santa Cruz, CA, USA) and HSP 90 (4F10) (sc-69703; Santa Cruz Biotechnology Inc.) antibodies were used at 1:400 diluted in TBST + 5% skim milk, while the secondary goat-anti-mouse IgG-HSP antibody (sc-2005; Santa Cruz Biotechnology Inc.) was diluted at 1:1000. Primary antibodies were incubated overnight at 4°C and the secondary antibody was incubated for 1 h at room temperature. Immobilon Western Chemiluminescent HRP Substrate (Millipore, Oakville, Ontario, Canada) was used for signal detection. Images were acquired using an AlphaImager Gel Documentation system with FluorChem HD2 software, version 3.4.0. Image manipulation was limited to adjustments to brightness and contrast of the entire image.

### Total RNA Isolation

Total RNA was extracted using the mirVana miRNA isolation kit (Ambion, Life Technologies, Carlsbad, CA, USA) according to the manufacturer’s instructions. Thirty microlitres of elution solution were used to resuspend the pellet and quantity was determined by NanoDrop spectrophotometry. Similar quantities of RNA were isolated from Day 1 and Day 5 HKL EVs, indicating that both populations of cells secrete similar amounts of EV cargo. Samples were sent to the Atlantic Cancer Research Institute (Moncton, New Brunswick, Canada) for library preparation and sequencing.

### Library Preparation and miRNA Sequencing

RNA quality was assessed on a 5200 fragment analyzer (Agilent Technologies, Santa Clara, CA, USA) using the HS RNA assay and the HS small RNA assay (Agilent Technologies). Eight small RNA libraries were prepared using the Clean Tag Small RNA library prep kit following manufacturer recommended conditions (TriLink Biotech, San Diego, CA, USA). Ion Torrent specific RT primer and barcodes were used during the library construction. Barcoded cDNA libraries were subjected to double size selection (150–200 bp) using Ampure XP beads (Beckman Coulter, Mississauga, Ontario, CA) to enrich for miRNA transcripts. The quality of each library was analyzed using a D1000 assay on TapeStation 2200 (Agilent Technologies, Mississauga, Ontario, Canada). Libraries were equally pooled at a loading concentration of 7 pM and amplified onto Ion Sphere™ Particles (ISP) using the Ion PI Hi-Q™ OT2 kit (Life Technologies). The ISP enriched library was sequenced using the Ion Proton (ThermoFisher).

### Data Processing

The raw sequencing fastq files are deposited in NCBI’s Gene Expression Omnibus (GEO) under the identifier GSE143360 (accession numbers can be found in **Supplementary Table 1**). The adapter sequences were trimmed and size filtered (to remove reads shorter than 18 nucleotides (nts) or longer than 25 nts) using the Cutadapt Python Package (v.1.13). The sequence reads were mapped to a reference index consisting of all known mature miRNAs in Atlantic salmon (including the Atlantic salmon miRNAs in miRbase) (58, 59) using STAR aligner software (v2.4.2b). A complete overview of the unique mature Atlantic salmon miRNA sequences in this reference index can be found in Woldemariam et al. (58). The reference index is the current updated version of the Atlantic mature miRNAome previously provided to miRbase in 2013 (59). The alignment files were further processed in R using featureCounts (60) to produce count matrices that were used as input in the R package DESeq2 to determine miRNAs that were significantly differentially enriched (DE) in Day 1 and Day 5 (61).

### RT-qPCR Analysis of miRNA Expression

To validate the miRNA sequencing results, the experiment was repeated with a different group of Atlantic salmon. Total RNA was isolated using the mirVana kit, as above. cDNA was synthesized using the miScript II RT Kit (Qiagen, Hilden, Germany), as per the manufacturer’s instructions, with 100 ng of total RNA in 20 μl reactions. The sequences of the mature miRNAs of interest were used as forward specific primers (**Supplementary Table 2**) while a universal primer, provided by the miScript SYBR Green PCR Kit (Qiagen), was used as a reverse primer. Three-fold, 5-point standard curves of pooled cDNA from cultured HKLs were used to assess the quality of all miRNA primers, with the exception of ssa-miR-155-5p and ssa-miR-146a-5p, where a 4-point standard curve was used. RNA from cultured HKLs was used for primer quality control instead of RNA from HKL EVs due to the very low amount of RNA obtained from isolated EVs. The efficiencies of the primers ranged from 78.3% to 116.5%. As the miRNA primer is the same size of the miRNA, there is no way to improve the efficiency of the primer. The geometric mean of the two chosen normalizers (ssa-miR-30b-5p and ssa-miR-142-3p) showed stability between the two sample groups (i.e. average geometric mean of normalizers’ Ct less than 0.25 between the two groups). Mature miRNAs are extremely robust and the common methods to measure RNA quality cannot be used to judge the degree of degradation of mature miRNAs with an average size of 22 nts (62). However, the fact that the miRNAs applied as normalizers showed good agreement between the Day 1 group and the Day 5 group suggests that the miRNA was not degraded. Each reaction was run in duplicate and was composed of 12.5 μl of 2× QuantiTect SYBR Green PCR Master Mix, 2.5 μl of 10× miScript Universal Primer, 2.5 μl specific forward primer (10 μM), 5 μl RNase-free water (Qiagen), and 2.5 μl of diluted cDNA template representing 5 ng of input total RNA. The PCR program consisted of one cycle of 95°C for 15 min, and 40 cycles of 94°C for 15 s, 55°C for 30 s and 70°C for 30 s, followed by a final melting point analysis, on a 7500 Fast Real-Time PCR System (Applied Biosystems). Microsoft Excel was used to determine the relative quantity (RQ) values of each miRNA relative to the average delta Ct of the control miRNA (Day 1 samples) using the comparative Ct method (63), with the assumption of 100% efficiency of the primers.

### Statistical Analysis

DE miRNAs were identified by comparing the Day 1 group to the Day 5 group (n=4 from each experimental condition) applying DESeq2 as previously described in 2.9. For RNA-seq, miRNAs were considered to be statistically DE if they had a Benjamini-Hochberg adjusted p-value of <0.05, base mean read counts >20 and log_2_ fold-change of >|1|. The average normalized read count in Day 1 cells and Day 5 cells from the DESeq2 analysis was used to reveal the miRNA diversity and abundance in EVs released from Day 1 and Day 5 HKLs. A paired Student’s T-test was used to determine statistically significant differences between Day 1 and Day 5 RT-qPCR samples using the Prism package v 8.0 (GraphPad Software Inc., La Jolla, CA). Genesis software (Rockville, Maryland, USA) was used for the hierarchical clustering of median centred normalized counts of DE miRNAs using the Pearson correlation and complete linkage clustering.

### *In Silico* Prediction of Target Genes and Gene Pathway Enrichment Analysis

The putative target genes of the DE miRNAs were predicted using the target prediction tool RNAhybrid 2.2 (64). The parameters applied in the *in silico* prediction ensured that only matches with perfect seed complementarity and high base-pairing stability were returned to minimize false positives. The settings were: helix constraint 2-8, no G: U in seed and minimum free energy threshold -18 kcal/mol as described in Andreassen et al. (38) and the predictions were against 3’UTRs from all Atlantic salmon mRNA transcripts (NM entries) in the Refseq database in GenBank (https://www.ncbi.nlm.nih.gov/). Gene symbols and gene IDs of the predicted target transcripts were extracted from the Universal Protein Resource (UniProt) database https://www.uniprot.org/ (65). The gene pathway enrichment analysis was carried out as in Woldemariam et al. (36) using Enrichr (66, 67) to identify significantly enriched pathways and gene ontology (GO) terms using the predicted miRNA targets as input. GO terms and pathways with adjusted p-value < 0.05 were considered as significantly enriched.

## Results

### Atlantic Salmon Adherent HKLs Release Extracellular Vesicles During In Vitro Culturing

**Figure 1:**
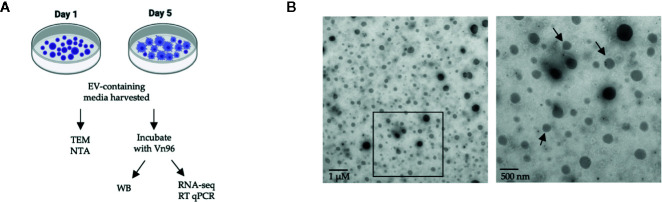
Confirmation of extracellular vesicle (EV) release from Atlantic salmon HKLs. **(A)** Diagram of experimental workflow. **(B)** Transmission electron microscopy (TEM) images of EVs released into cell culture media by Day 1 HKLs (magnification 2700x and 6500x respectively; size of scale bar indicated on image). Area within the square of the left image is magnified in the right image. Arrows are pointing to double membranes. TEM images representative of n=3. NTA, Nanoparticle tracking analysis; WB, Western blot; RNA-seq, RNA-sequencing; RT-qPCR, reverse transcription quantitative polymerase chain reaction.

EVs were characterized according to the Minimal Information for Studies of Extracellular Vesicles 2018 (MISEV2018) guidelines using TEM, NTA, and Western blotting for the EV protein HSP90 (68). We recognize that detection of additional protein markers is suggested in the MISEV2018 guidelines; however, the wide testing of available antibodies for the canonical EV markers that cross-react with Atlantic salmon proteins is beyond our present capacity. The workflow for this experiment can be found in **Figure 1A**. To confirm that putative EVs were released from HKLs into the culture media, the culture media was analyzed by TEM. Round, double-membraned structures of variable sizes were observed in the culture media (**Figure 1B**), confirming that the HKLs secrete EVs.

**Figure 2:**
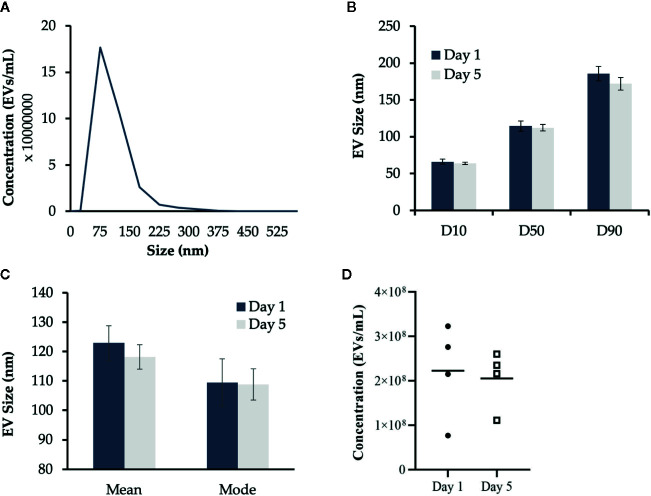
Characterization of Atlantic salmon extracellular vesicle (EV) size and quantity. Cell culture media containing EVs released from adherent Atlantic salmon HKLs was analyzed using nanoparticle tracking analysis (NTA). Five videos were captured per sample and results were reported as an average of the five videos. **(A)** Representative histogram of EV size profile **(B)** EV size distribution D10 (diameter where 10% of the population lies below the D10), D50 (diameter where 50% of the population lies below D50), and D90 (diameter where 90% of the population lies below D90) for EVs released at Day 1 and Day 5. Data reported as average mean +/- SE. **(C)** Mean size and mode size (+/- SE) of EVs released from Day 1 and Day 5 HKLs. **(D)** Concentration of EVs released from Day 1 and Day 5 HKLs. Scatterplots show data from individual fish (average of five videos); n=4; no statistical differences were observed as determined by a paired Student’s T-test.

Our previous research determined that Day 1 HKLs are primarily “monocyte-like” while Day 5 HKLs are primarily “macrophage-like” (47). Therefore, the concentration and size of EVs released from Day 1 and Day 5 HKLs were analyzed by NTA (**Figure 2**); an accurate and precise method to measure both size and concentration, as opposed to TEM. EVs from Day 1 HKLs had a mean size of 122.9 nm, a mode size of 109.5 nm and a range of 109.0–137.6 nm. EVs from Day 5 HKLs had a mean size of 118.2 nm, a mode size of 108.8 nm and a range of 107.6–127.4 nm (**Figures 2B, C**). The average concentration of EVs from Day 1 HKLs was 2.23x10^8^ ± 1.08x10^7^ EVs/ml, while the average concentration of EVs from Day 5 HKLs was 2.06x10^8^ ± 8.73x10^6^ EVs/ml (**Figure 2D**). Overall, there was no significant difference in the size (p=0.6363) or concentration (p=0.8162) of EVs released from Day 1 HKLs compared to EVs released from Day 5 HKLs, as determined by a paired Student’s T-test.

### Confirmation of EVs Derived From Atlantic Salmon Adherent HKLs by Vn96 Isolation

**Figure 3:**
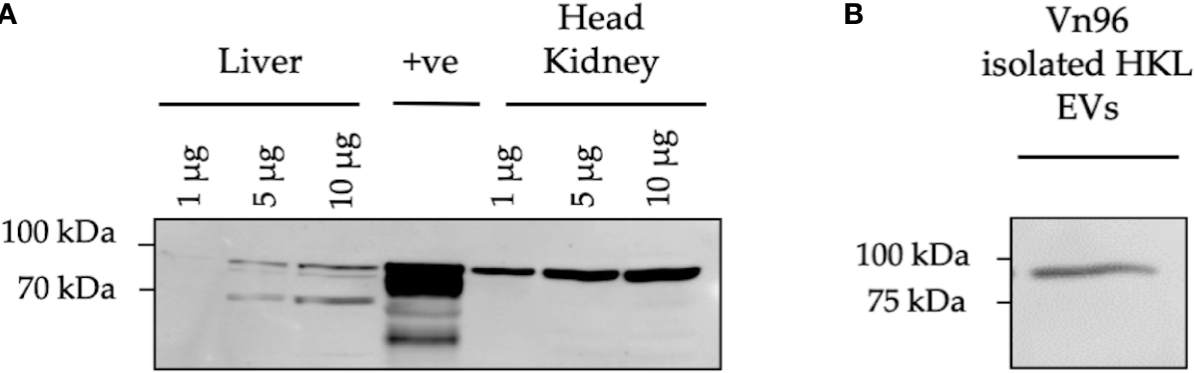
HSP90 protein expression in Atlantic salmon HK, liver, and HKL derived extracellular vesicles (EVs). **(A)** Protein lysates from Atlantic salmon liver and head kidney tissue at 1, 5, and 10 μg were tested for cross-reactivity with anti-mouse HSP90. Wehi-231 murine B cells were used as a positive control (+ve). **(B)** HSP90 expression in Vn96 isolated EVs derived from Day 1 HKL culture media.

In this study, the Vn96 peptide was used to isolate EVs for small RNA-sequencing (RNA-seq). Vn96 can bind to several distinct HSPs, found on the exterior of the EV, from multiple species including human, canine, rodents (mouse and hamster), bovine, and Chinook salmon (56). However, it was not previously demonstrated that Vn96 can bind to HSPs on EVs from Atlantic salmon. Therefore, we first confirmed that Vn96 binds to EVs derived from Atlantic salmon HKLs based on the detection of HSPs using Western blotting. Due to the limited availability of commercial Atlantic salmon antibodies (Abs), we first sought to confirm cross-reactivity of anti-mouse Hsc70 and anti-mouse HSP90 Abs with Atlantic salmon head kidney and liver lysates, using the Wehi-231 murine B-cell line as a positive control (69). We found that the anti-mouse Hsc70 Ab did not cross-react with lysates from Atlantic salmon (data not shown). However, the anti-mouse HSP90 Ab detected a protein of the same molecular weight in both Atlantic salmon and mouse (**Figure 3A**). Using the anti-HSP90 Ab, we confirmed that the Vn96 peptide successfully enriched HSP90-positive EVs from Atlantic salmon HKL culture media since one protein species at the expected size of 90 kDa was detected (**Figure 3B**).

### RNA-Seq Identified 19 Differentially Packaged miRNAs in Day 1 and Day 5 HKL EVs

**Figure 4:**
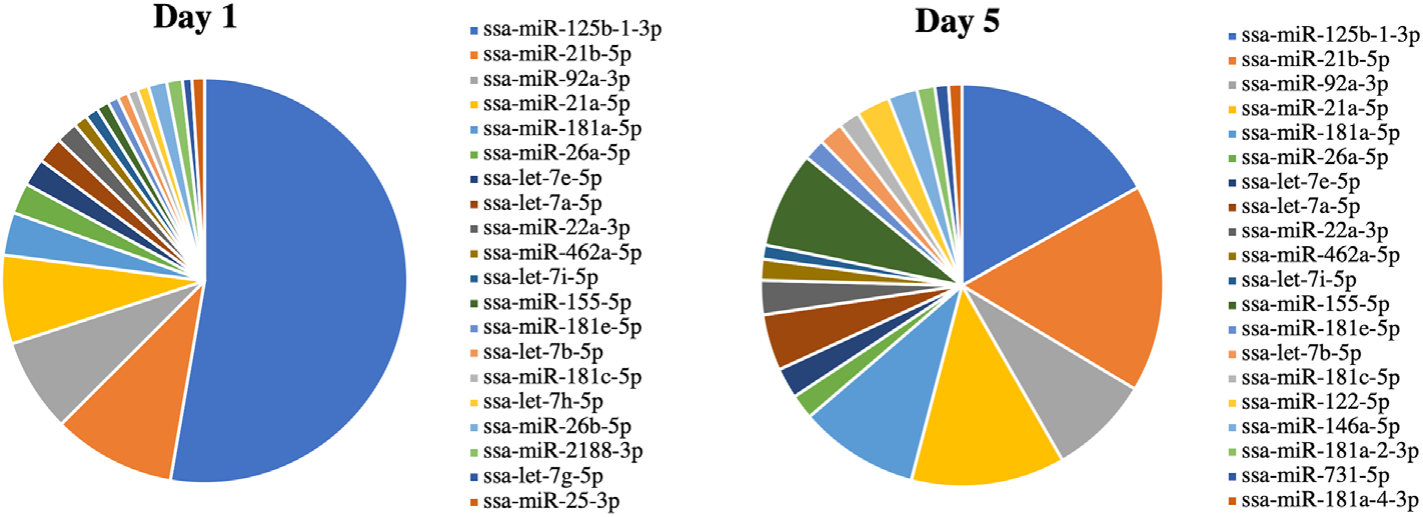
miRNA abundance (average normalized read counts) in extracellular vesicles (EVs) released from Day 1 and Day 5 Atlantic salmon HKLs. The top 20 most abundant miRNAs in Day 1 and Day 5 EVs are shown.

RNA-seq was used to examine the miRNAs packaged in Vn96-isolated EVs released from Day 1 and Day 5 HKLs. The number of reads mapped to miRNAs ranged from 22,364 to 61,094 (**Supplementary Table 1**). A total of 479 miRNAs were detected in either Day 1 or Day 5 HKL EVs (**Supplementary Table 3**). However, most of these consisted of very low counts. Twenty-two and thirty miRNAs with an abundance of more than 0.5% were identified in Day 1 and Day 5 groups, respectively, while six and 10 miRNAs had an abundance of more than 2% in Day 1 and Day 5 groups, respectively (**Supplementary Table 4**). Interestingly, ssa-miR-125b-1-3p was the most abundant mature miRNA in both groups representing 45% and 14% of all mature miRNAs in Day 1 and Day 5 EVs, respectively (**Supplementary Table 4**), while representing 52.7% and 16.9% of the top 20 most abundant miRNAs in Day 1 and Day 5 EVs, respectively (**Figure 4**).

**Figure 5:**
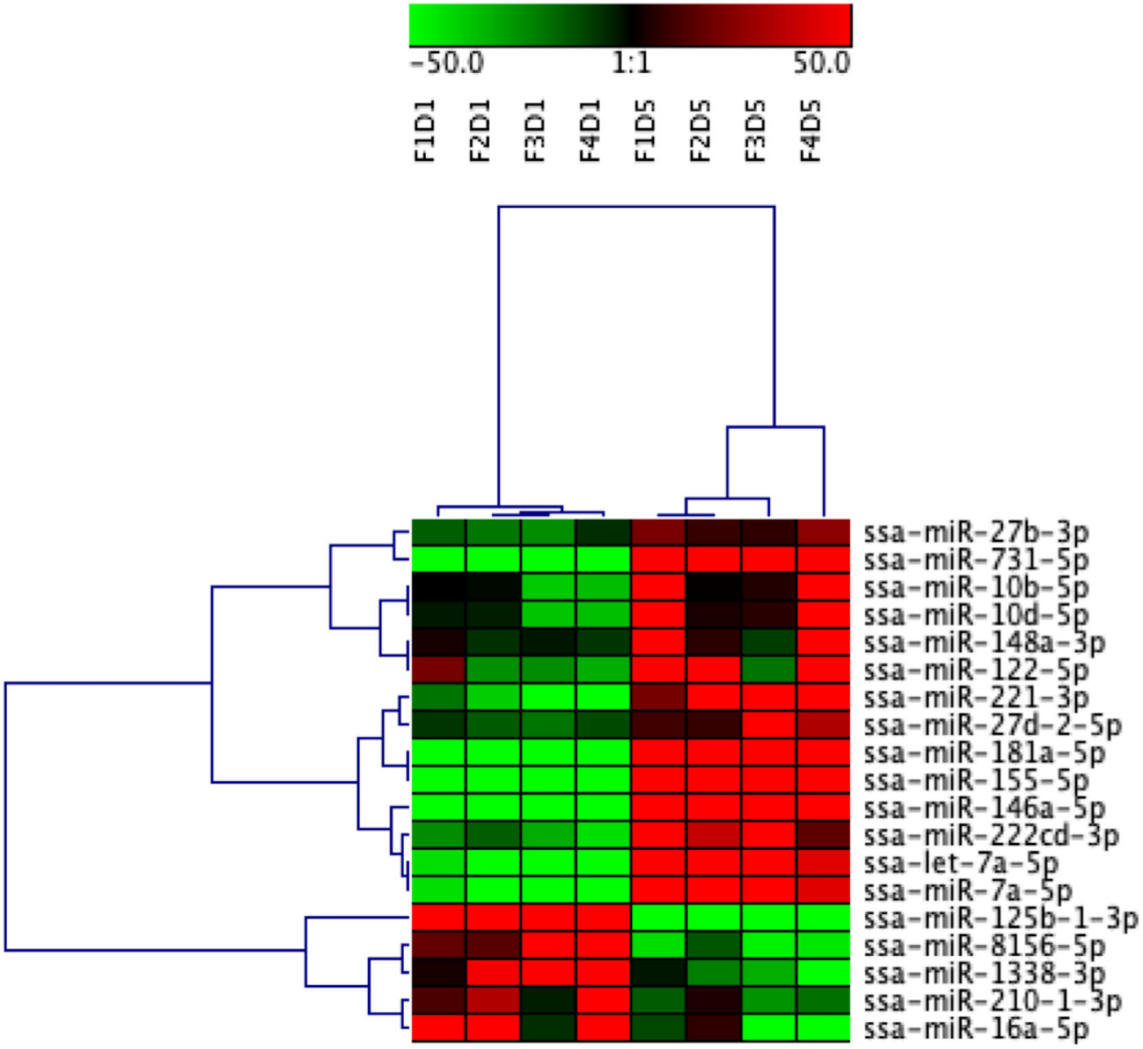
Heatmap illustration and hierarchical clustering analyses of differentially enriched miRNAs packaged in extracellular vesicles (EVs) released from Day 1 and Day 5 HKLs. The heatmap represents the normalized counts of DE miRNAs in EVs released from Day 1 HKLs and EVs released from Day 5 HKLs in each individual fish. miRNA normalized counts were median centred and clustered using Pearson correlation and complete linkage hierarchical clustering. Red indicates higher counts and green indicates lower counts. Integer adjusted to a maximum of 50 and a minimum of -50. F indicates fish number; D indicates Day 1 or Day 5 (i.e. F1D1 is Fish 1 Day 1).

DE miRNAs between EVs released from Day 1 HKLs compared with EVs released from Day 5 HKLs were analysed by DESeq2. There were 19 DE miRNAs in Day 1 HKL EVs compared with Day 5 HKL EVs (**Table 1**). Thirteen miRNAs were more abundant in EVs released from Day 5 HKLs, while six miRNAs were less abundant in EVs released from Day 5 HKLs (**Table 1**). Several of the most abundant miRNAs were also DE including ssa-miR-125b-3p, ssa-miR181a-5p, and ssa-miR-155-5p (**Table 1**), while the highly abundant ssa-miR-21a and ssa-miR-21b were not significantly DE despite a rather large difference in percentage of these miRNAs between Day 1 and Day 5 EVs (**Figure 4**). Hierarchical clustering analyses of the DE miRNA based on normalized counts showed that all samples from Day 1 HKL EVs clustered together and all samples from Day 5 HKL EVs clustered together, indicating the two groups represent distinct sub-populations (**Figure 5**). However, we also observed a large variation in normalized read counts within groups for several miRNAs (e.g. ssa-miR-148a-3p, **Supplementary Table 3**) indicating that there was substantial variability in abundance of certain miRNAs within each of the two groups. Given the relative paucity of available data involving investigations of fish-derived EVs and their molecular cargo, considerable potential remains for optimizing future studies.

**Table 1:** Differentially enriched miRNAs in Day 5 compared with Day 1 extracellular vesicles (EVs) released from Atlantic salmon HKLs.

miRNA^a^	baseMean	log_2_ Fold-change^b^	Adjusted p-value	Mature sequence 5-3’
**Upregulated**				
ssa-miR-122-5p	392.55	4.95	0.001	TGGAGTGTGACAATGGTGTTTG
ssa-miR-155-5p	1235.36	2.89	<0.001	TTAATGCTAATCGTGATAGGGGT
ssa-miR-146a-5p	384.47	2.63	<0.001	TGAGAACTGAATTCCATAGATGG
ssa-miR-148a-3p	47.68	2.46	0.011	TCAGTGCATTACAGAACTTTGT
ssa-miR-27d-2-5p	30.70	1.80	0.006	AGGACTTAGCACACATGTGAACA
ssa-miR-731-5p	207.06	1.54	<0.001	AATGACACGTTTTCTCCCGGATT
ssa-miR-10d-5p	119.34	1.47	0.027	CACCCTGTAGAACCGAATTTGT
ssa-miR-10b-5p	122.16	1.46	0.032	TACCCTGTAGAACCGAATTTGT
ssa-miR-181a-5p	1866.45	1.41	0.026	AACATTCAACGCTGTCGGTGAGT
ssa-miR-27b-3p	28.37	1.37	0.027	TTCACAGTGGCTAAGTTCTGC
ssa-miR-221-3p	204.17	1.34	0.006	AGCTACATTGTCTGCTGGGTTTC
ssa-miR-222cd-3p	93.62	1.18	0.009	AGCTACATCTGATTACTGGGTCA
ssa-let-7a-5p	950.56	1.02	0.030	TGAGGTAGTAGGTTGTATAGTT
**Downregulated**				
ssa-miR-16a-5p	143.42	-1.12	0.053	TAGCAGCACGTAAATATTGGAG
ssa-miR-210-1-3p	35.34	-1.47	0.038	CTGTGCGTGTGACAGCGGCT
ssa-miR-1338-3p	88.38	-1.52	0.011	ATCTCAGGTTCGTCAGCCCATG
ssa-miR-7a-5p	35.86	-1.52	0.009	TGGAAGACTAGTGATTTTGTTGT
ssa-miR-125b-1-3p	10213.94	-1.72	0.005	ACAGGTGAGGTCCTCGGGAA
ssa-miR-8156-5p	61.01	-2.64	0.001	GTCCTGACTGTCCTGACTGTC

a The names are in a few cases with different lettered/numbered suffixes than in miRBase as several mature family members are identical.

b Negative fold-change values are down-regulated in Day 5 compared with Day 1; positive fold-change values are up-regulated in Day 5 compared with Day 1.

### RT-qPCR Analysis Confirmed miRNA Abundance in Day 1 and Day 5 HKL EVs

**Figure 6:**
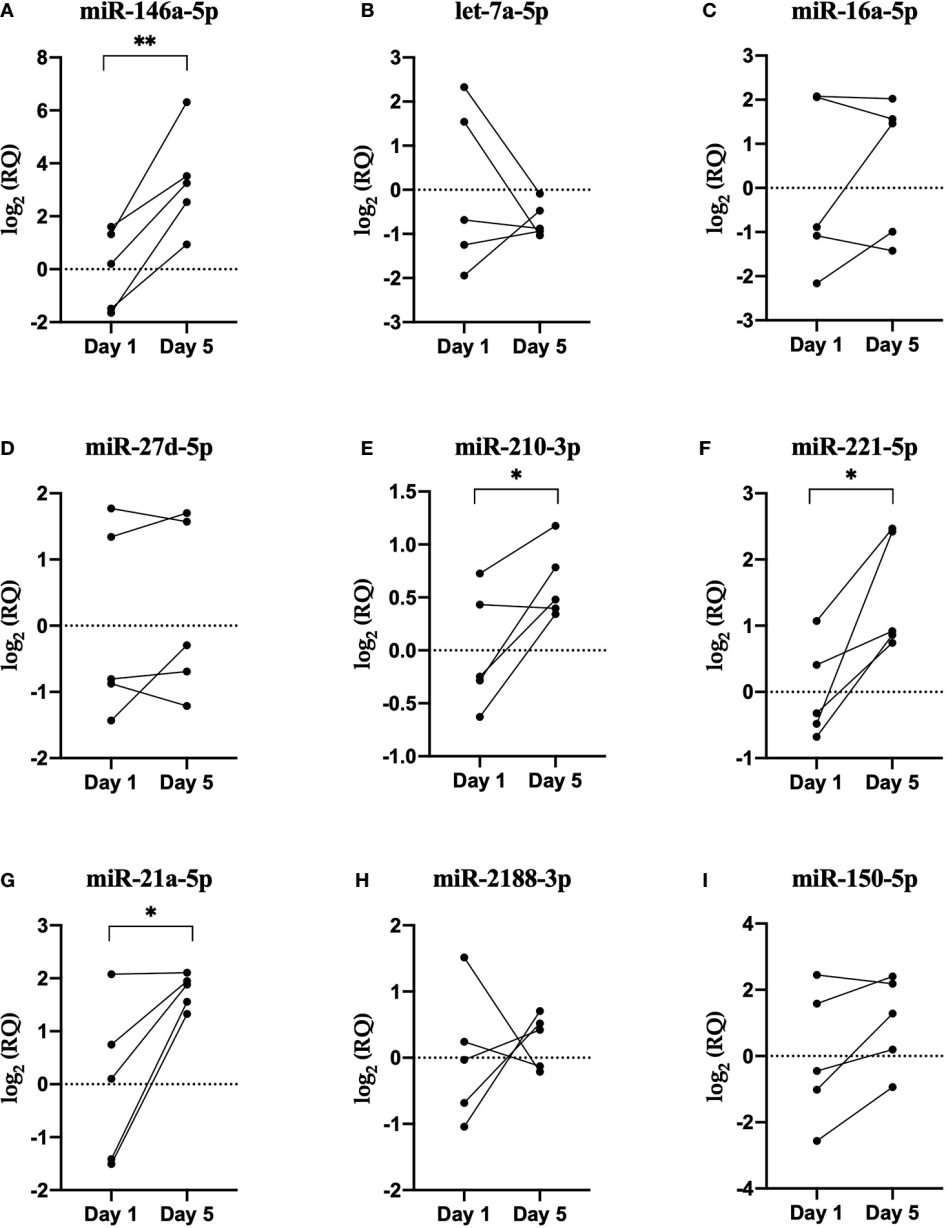
RT-qPCR results. Scatterplots of the relative quantity (RQ) values of miRNAs determined by RNA sequencing to be DE between EVs released from Day 1 and Day 5 HKLs. Scatterplots show individual data with lines connecting data point from each individual fish, n=5. *p < 0.05; **p < 0.01. **(A)** miR-146a-5p **(B)** let-7a-5p **(C)** miR-16a-5p **(D)** miR-27d-5p **(E)** miR-210-3p **(F)** miR-221-5p **(G)** miR-21a-5p **(H)** miR-2188-3p **(I)** miR-150-5p.

Five new Atlantic salmon (i.e. different from the salmon used for RNA-seq) were used for the RT-qPCR analysis. Nine miRNAs were chosen for RT-qPCR validation, along with two normalizer miRNAs (ssa-miR-30b-5p and ssa-miR-142a-3p) (**Figure 6**). A combination of upregulated and downregulated miRNAs was chosen for RT-qPCR, as well as miRNAs involved in vertebrate immune responses and macrophage function (31, 70, 71). In addition, we examined immune-relevant miRNAs that were identified by RNA-seq but not DE to confirm their presence in fish EVs: the fish-specific ssa-miR-2188-3p, as well as ssa-miR-21a-5p, ssa-miR-150-5p, and ssa-miR-221-5p, which are all virus responsive miRNAs in teleost fish, and involved in mammalian macrophage activation and/or differentiation (37, 72–75). All nine miRNAs that were detected by RNA-seq were also detected by RT-qPCR. However, only one miRNA, ssa-miR-146a-5p, showed the same differential expression found by RNA-seq in both direction and significance. One miRNA that was not identified as DE by RNA-seq (ssa-miR-21a-5p) was found to be DE by RT-qPCR. While ssa-miR-221-5p could not be analyzed by RNA-seq method due to low read numbers, the qPCR-method, being more sensitive, detected a significant increase in the low abundant 5p mature ssa-miR-221. A comparison of the sequencing and RT-qPCR results can be found in **Table 2**. The RT-qPCR results showed considerable biological variability between fish and miRNA expression. This variability is clear from the high standard error (SE) in many of the miRNAs examined *via* RT-qPCR and RNA-seq (**Table 2**) and is illustrated in the heat map of the RNA-seq data (**Figure 5**).

**Table 2:** Comparison of sequencing and RT-qPCR results^a^.

	RNA-seq							RT-qPCR				
	Average Normalized Count^b^		Standard Error		p-value^b^		p-value (padj)^c^	AverageLog_2_ RQ^d^		Standard Error		p-value^e^
miRNA	Day 1	Day 5	Day 1	Day 5				Day 1	Day 5	Day 1	Day 5	
miR-146a-5p	106.66	662.28	3.86	100.28	<0.001	<0.001		0.00	3.31	0.68	0.87	0.004
let7a-5p	627.33	1273.78	109.71	636.89	0.003	0.030		0.00	-0.68	0.82	0.18	0.437
miR-16a-5p	196.71	90.13	27.66	19.54	0.007	0.053		0.00	0.53	0.87	0.72	0.387
miR-27d-5p	13.62	47.79	2.31	11.63	0.001	0.006		0.00	0.22	0.62	0.61	0.455
miR-210-3p	51.95	18.72	11.67	5.29	0.005	0.038		0.00	0.64	0.25	0.16	0.033
miR-221-5p	0.00	3.77	0.00	1.89	0.531	n/a		0.00	1.48	0.32	0.39	0.033
miR-21a-5p	2096.99	3480.17	143.06	615.90	0.033	0.136		0.00	1.76	0.68	0.14	0.032
miR-2188-3p	372.12	112.00	147.83	46.94	0.017	0.377		0.00	0.26	0.43	0.15	0.691
miR-150-5p	22.30	13.69	4.60	0.98	0.015	0.355		0.00	1.03	0.90	0.63	0.079

a RNA-seq and RT-qPCR experiments were completed with two different groups of fish.

b As determined by a paired Student’s T-test.

c As determined by a paired Student’s T-test adjusted using the Benjamini-Hochberg method.

d Mean RQ of Day 1 was set to 1.0. Mean log_2_ relative quantity (RQ).

e As determined by a paired Student’s T-Test.

### Target Gene Prediction and Gene Pathway Enrichment Analysis

The *in silico* prediction of target genes showed that the 19 DE miRNAs could potentially target between 39 and 225 mRNA transcripts each. In total, there were 2,873 potential targets, however, as several DE miRNAs targeted the same transcripts, there were only 1,556 unique transcripts that were putative targets. The results from the *in silico* target prediction analysis for each of the DE miRNAs is given in **Supplementary Table 5**. Subsequent pathway analysis and GO term enrichment analysis did not result in any significant findings (**Supplementary Table 6**).

## Discussion

This study examined the miRNA cargo in EVs released from Atlantic salmon HKLs as they differentiated *in vitro*. Verification of EV isolation for study remains an evolving topic, and we have used the gold-standard approaches of TEM, NTA, and Western blot to verify the identity, isolation and quantity of these structures. By TEM we identified round, double-membraned structures consistent with previous reports on EV structure (76–78). Next, the size distribution and the concentration of EVs released from Day 1 and Day 5 HKLs were quantified and found to be consistent across time points *in vitro*. Using Western blot for HSP90, we then confirmed the presence of a canonical mammalian EV protein marker is also associated within Atlantic salmon EVs. Finally, we report the presence and potential differential packaging of miRNAs, including immune-related miRNAs, into EVs released from Day 1 HKLs and Day 5 HKLs using small RNA-seq and RT-qPCR. These studies provide some of the first evidence for the isolation and validation of EVs from Atlantic salmon, and therefore provide a starting point for future studies aimed at examining these EV cargos, such as miRNA, in greater detail.

### The Abundance Profile of the EV miRNAs and the Difference Between Day 1 and Day 5 Suggest They Have a Role in Macrophage Differentiation

The abundance of miRNAs in Day 1 EVs and Day 5 EVs show some striking differences to the abundance in monocyte-like HKLs (Day 1) and macrophage-like HKLs (Day 5) (47). The mature ssa-miR-125b-1-3p is the most abundant miRNA in both groups of EVs, while it is less than 0.01% of mature miRNAs in the cells. Likewise, ssa-miR-92a-3p, ssa-miR-181a-5p, and ssa-miR155-5p are much more abundant in EVs compared to their relative abundance in the cells at the same stage of differentiation [Supplementary Table 4 and Supplementary File 1 in (47)]. This indicates that they are selectively enriched in EVs as their abundance is not reflective of the general abundance in the cells. In addition, ssa-miR-125b-1-3p, ssa-miR-181a-5p, and ssa-miR155-5p also showed significant differences when comparing Day 1 EVs to Day 5 EVs. Together, this suggests that the miRNAs in the EVs serve particular functions. Altogether, the RNA-seq analysis identified 19 DE miRNAs packaged in Day 1 versus Day 5 HKL EVs. Most of these are conserved miRNAs (identical “seed” and very similar mature sequences in most vertebrates), and studies of miRNAs associated with macrophage differentiation and immune responses in other species may, therefore, shed light on their putative functions in Atlantic salmon.

The most abundant mature miRNA ssa-miR-125b-1-3p showed a large decrease from Day 1 EVs to Day 5 EVs. Interestingly, the miRNA-125 family is involved with immune system development and host defense [reviewed in (79)]. In particular, miR-125b expression is enriched in murine macrophages, compared to T-cells and B-cells (80). Overexpression of miR-125b in murine bone marrow cells induced a spread-like morphology with pseudopods and increased the protein expression of MHCII and the co-stimulatory molecules CD40, CD86, and CD80, indicating that miR-125b potentiates macrophage activation (80). Similarly, a study by Zhang et al. (2013) identified a decrease in miR-125b expression in M1 macrophages compared to M2 macrophages (73). In addition to identifying a decrease in miR-125b in Day 5 cells compared to Day 1 cells, our results identified monocyte to macrophage differentiation protein (*paqrb)* as a potential target of miR-125b. It is possible that the role of miR-125b in macrophage differentiation and function may be species specific. However, further experiments are required to determine this.

The sequencing results of this study found an increase in ssa-let-7a in Day 5 EVs. However, the RT-qPCR results did not find a significant difference in let-7a incorporation between Day 1 and Day 5 EVs which may in part be due to a different group of salmon being used for RNA-seq and RT-qPCR. Let-7a miRNA expression is induced by LPS stimulation in human primary macrophages (81). In addition, overexpression of let-7a in human THP-1 macrophages attenuated the increase of pro-inflammatory TNF-α and IL-6 mRNA levels following LPS stimulation (82). These studies suggest a role for let-7a in macrophage function and inflammation.

RNA-seq identified increased incorporation of ssa-miR-155-5p in Day 5 EVs compared to Day 1 EVs. While we did not include ssa-miR-155-5p in the RT-qPCR study, our previous work found an increase in ssa-miR-155-5p in Day 5 HKLs compared to Day 1 HKLs by RNA-seq and RT-qPCR (47). In several other species, miR-155 is involved in macrophage differentiation and function. The addition of exosomes loaded with miR-155 inhibitor to murine RAW macrophages resulted in decreased LPS-induced TNF-α protein levels (83). In mammalian macrophages, miR-155 expression is increased following infection with *Listeria monocytogenes* or *Mycobacterium avium* and stimulation with both LPS and poly(I:C) (84, 85). Similarly, stimulation of macrophages isolated from the fish species ayu (*Plecoglossus altivelis*) with *Vibrio anguillarum* increased miR-155 expression while overexpression of miR-155 increased the expression of pro-inflammatory cytokines and decreased the expression of anti-inflammatory cytokines (86). The results of these studies, in addition with the results of this current study, suggest that miR-155 may be a marker of immune response in both EVs and miRNAs.

### Increased ssa-miR-146a Incorporation in Day 5 HKL EVs Compared to Day 1 HKL EVs

Despite substantial biological variability between individual fish, this study validated the increased packaging of ssa-miR-146a-5p in EVs derived from Day 5 HKLs compared to EVs from Day 1 HKLs by both RNA-seq and RT-qPCR. MiR-146a plays a role in macrophage differentiation, activation and function in several species including some fish species (74, 87–91). However, to date, no studies have identified the presence of miR-146a in fish EVs. A study by Song et al. (92) found that human mesenchymal stem cells (MSCs) stimulated with the pro-inflammatory cytokine Il-1β produced exosomes that transferred miR-146a to macrophages where it induced the downregulation of M1 markers and upregulation of M2 markers, suggesting it has a role in macrophage polarization (92). In other studies, miR-146a -/- knockout mice injected with miR-146a-containing exosomes had reduced TNF-α and Il-6 serum levels following LPS injection, compared to mice injected with miR-146a deficient exosomes, demonstrating that exosomal miR-146a can also play a role in moderating the inflammatory response (29).

At the cellular level, miR-146a is induced by PU.1, a transcription factor that stimulates the differentiation of hematopoietic stem cells (HSCs) into lymphoid-myeloid progenitors (90). In a mouse transplant model, forced expression of miR-146a directed the differentiation of HSCs into peritoneal macrophages. Congruently, preventing miR-146a function in a zebrafish model inhibited the formation of macrophages (90). Infection of human primary monocytes and the human monocytic cell line THP-1 with bacterial pathogens such as *Salmonella* serovar Typhimurium DT104 and *Mycobacterium avium*, and infection of murine bone marrow-derived macrophages (BMDMs) with *Listeria monocytogenes* increased miR146a expression, suggesting that miR-146a is involved in regulating macrophage response to infection, as well as their differentiation (84, 85, 93). In addition, its expression is increased in the Atlantic salmon head kidney following formalin-killed typical *Aeromonas salmonicida* or poly(I:C) injection (94). Overexpression of miR-146a in grouper macrophages promoted Singapore grouper iridovirus (SGIV) propagation while inhibition of miR-146a decreased virus production (88). In our previous study, miR-146a was upregulated in Day 5 HKLs compared to Day 1 HKLs (47). Therefore, we propose that since miR-146a is DE in Day 1 and Day 5 EVs and HKLs, it may be an indicator for the presence of macrophage cells and/or macrophage activation and function.

### The Highly Abundant miR-21a Is Associated With Macrophage Activation in Vertebrates

While RNA-seq did not identify a significant difference in the incorporation of ssa-miR-21a-5p, RT-qPCR analysis identified a significant increase in ssa-miR-21a-5p in Day 5 HKL EVs. Again, this may in part be due to a different group of fish being used for RNA-seq and RT-qPCR. At the cellular level, miR-21a is involved in macrophage activation and immune response in both mammals and fish [33,72,73]. MiR-21 deficient mice had decreased expression of M1 macrophage markers and enhanced expression of M2 markers, while transfection of a miR-21 mimic enhanced M1 markers and decreased M2 markers (95). Stimulation with LPS, poly(I:C) or *V. anguillarum* upregulated expression of miR-21 in cultured macrophages from miiuy croaker (33, 96). Inhibiting miR-21 in miiuy croaker macrophages increased the expression of inflammatory cytokines (*tnfa*, *il6)* and antiviral genes (*mx1, isg15*), suggesting a role for miR-21 in fish macrophage function (96). Our previous work identified miR-21a as the most abundant miRNA in both Day 1 and Day 5 HKLs, as well as upregulated in Day 5 HKLs compared to Day 1 HKLs (47).

M1 macrophages are broadly considered to be pro-inflammatory, whereas M2 macrophages are considered anti-inflammatory (42, 43). During an immune response, there is an increase in macrophage number and activity (97). The enrichment of miR-21a in Day 5 HKL EVs is, therefore, suggestive of differentiation associated with M1 polarization (unlike miR-146a) and may be useful as an EV biomarker for evaluating pro-inflammatory vs. anti-inflammatory responses in Atlantic salmon. Future studies examining the miRNA packaged in EVs following pathogen exposure, or stimulation with M1 (i.e. IFNγ) or M2 (i.e. IL-4 or IL-13) cytokines, are required to test this hypothesis.

Interestingly, it has been previously reported that the abundance of miR-21 in Atlantic cod EVs is responsive to changing environmental conditions. Exosomes isolated from Atlantic cod sera contained higher levels of miR-21 in fish reared at 9°C compared to fish reared at 4°C, suggesting that EV miR-21 may be a biomarker for exposure to environmental stress (9). In addition, there were significantly less EVs in the serum of cod reared at 9°C further suggesting EV biogenesis is linked to environmental conditions (9).

### Teleost-Specific ssa-miR-2188 and ssa-miR-731 Are Present in HKL EVs

MiR-731 and miR-2188 are teleost-specific, immune-responsive miRNAs (31, 38, 39). This study is the first to identify miR-731 and mir-2188 in fish EVs. The sequencing results of this study found a significant increase of miR-731 in Day 5 EVs compared to Day 1 EVs and a decrease of miR-2188 in Day 5 EVs, although the decrease of miR-2188 was not significant. In several fish species including Atlantic salmon, miR-731 is upregulated in response to both viral and bacterial challenges (31, 36, 38, 41). Loss of the miR-462-731 cluster in zebrafish decreased erythroid cell numbers, and myeloid cell expansion, suggesting a role for miR-731 in regulating hematopoiesis (98). Interestingly, the PU.1 motif and several IRF-binding motifs are upstream of the miR-462/731 miRNA gene indicating that these mature miRNAs are important in hematopoietic stem cell differentiation and immune response (38). In Atlantic salmon and in olive flounder (*Paralichthys olivaceus*), miR-2188 expression decreased in cardiac tissue following salmonid alphavirus (SAV) infection and decreased in the head kidney following viral hemorrhagic septicemia virus (VHSV) infection, respectively (34, 38). Conversely, miR-2188 expression increased in Atlantic cod HKLs following 48 and 72 h of poly (I:C) stimulation, while its expression was significantly downregulated in unstimulated cod HKLs at 72 h compared to cells cultured for 12 and 24 h (39).

### Discrepancies Between RNA-Seq and RT-qPCR Data

EV biogenesis is known to generate a diverse, heterogeneous population of vesicles. Previous studies have shown that individual EVs may vary considerably with respect to the biomolecules they incorporate, but that the population as a whole may be representative of a particular cell type or physiological state (99, 100). However, validation of results using complementary techniques remains technically challenging. EVs have a small internal lumen space and packing volume (99, 101, 102) and therefore a limited number of molecules are available for analysis in any given EV isolation. Due to the low concentration of RNA that could be isolated from the EVs, our RNA-seq and RT-qPCR analyses were performed on independent biological samples (i.e. different groups of Atlantic salmon). While our RT-qPCR analysis validated the presence of all miRNAs identified by RNA-seq, we could only corroborate a significant change of one of the miRNAs (ssa-miR-146a-5p).The use of a different group of fish for RNA-seq and for RT-qPCR may in part account for the variability between sequencing and RT-qPCR results. Despite these limitations, it is particularly noteworthy that two independent groups of fish showed the inclusion of the same miRNA species, suggesting that these miRNAs reflect an accurate depiction of the underlying cellular biology. In addition, several of the miRNAs identified in this study are immune-relevant in both mammals and fish. The RT-qPCR results exhibited high biological variability as observed in RNA-seq, but together these data suggest that differential packaging of miRNAs into EVs, including miRNAs involved in the immune response and macrophage activation, is a feature of Atlantic salmon HKL differentiation. Future studies will seek to improve power by performing validation experiments on a larger cohort of specimens or minimize intra-assay biological variability by using pooled populations of EV material.

HSPs appear to be the best conserved markers of EVs (103) and are present essentially ubiquitously. A study in rainbow trout found increased HSP70 protein expression in exosomes released from hepatocytes following a 1 h heat shock (10). However, cortisol treatment significantly reduced the expression of HSP70 in hepatocyte released exosomes (10). Nevertheless, since Vn96 binds to multiple HSPs, changes to individual proteins will not likely impact the overall number of EVs isolated when cells are similarly healthy. We found that cells in both Day1 and Day 5 HKL cultures were similarly healthy based on visual observations of adherent cultures where there was no change in cell debris seen, the observation that EV concentration and size were similar, and similar amounts of RNA were extracted. In addition, our previous study found that similar amounts of RNA were extracted from the cells and there was an increase in phagocytic ability at Day 5 (47). Therefore, we conclude that Vn96-based isolation is a sound approach. In addition, previous experiments in our lab have found that Vn96 can extract EVs in multiple sequential incubations which suggests that the one incubation performed here is not enough to saturate the capacity of the Vn96. It remains to be determined if different quantities of EVs from healthy compared to highly stressed cells are isolated by Vn96.

### *In Silico* Analysis of DE miRNAs Identified Potential Targets That Are Macrophage and Immune Relevant

We performed *in silico* target prediction analysis to identify putative targets of the DE miRNAs in Day 1 and Day 5 HKL EVs. The identified putative targets included transcription factors, lipid-related genes and immune-related genes that are associated with macrophage function and immune response. Specific examples include the transcription factors *klf2* (a putative target of miR-222cd-3p and miR-221-3p) and *gata3* (a putative target of miR-146a-5p and miR-125b-1-3p). *Klf2* is a negative regulator of monocyte activation and function while *gata3* is involved with the regulation of M2 macrophages (104–106). Lipid-related genes such as fatty acyl-CoA reductase (*facr1*; miR-27b-3p, miR-221-3p, miR-222cd-3p), sterol regulatory element-binding proteins (*srebps*; miR-731-5p, miR-27b-3p) and delta-6 fatty acyl desaturase (*d6fadc* or *fads2;* miR-181a-5p) were also identified as putative targets. In mammals, the lipid-related transcriptome changes dramatically during monocyte to macrophage differentiation, including the levels of *facr1, srebps* and *fads2*, suggesting they play a role in macrophage differentiation and/or function (107, 108). Finally, several immune and inflammation-related genes were also potential targets, including *tnfa* (miR-16a-5p), viperin (alias *rsad2*; miR-222cd-3p and miR-221-3p), granulin (miR-155-5p), *irfg* (miR-27b-3p), and the transcription factors *irf4, irf7* (miR-731-5p) and *irf9* (miR-8156-5p). In addition, monocyte to macrophage differentiation protein (*paqrb*) was a target of the most abundant miRNA (ssa-miR-125b-3p) which was also significantly DE in Day 1 vs Day 5 HKL EVs. It is important to note that many of these targets may likely be false positives (109) and functional assays, such as manipulation of the miRNA of interest, are necessary to prove a potential target.

### Further Studies Are Needed to Elucidate the Function of miRNAs in Fish EVs

Several studies have demonstrated the transfer and uptake of miRNA-containing EVs from multiple cells types, including immune cells [reviewed in (3, 110)]. EV transfer of miRNAs regulates gene expression in the target cell, thereby modulating its function (110). For instance, human monocytes release miR-150 containing EVs which are taken up by endothelial cells prompting cell migration; macrophage-derived EVs transfer miR-233 to monocytes inducing cell differentiation; and T cells release miR-335-containing EVs that are taken up by APS, modulating gene expression (28, 111, 112). The target cells and subsequent effects of HKL-derived EVs on these cells remain unexplored. We identified several immune-related miRNAs packaged in EVs released from HKLs. It is possible that these EVs target other immune cells to regulate their response during both health and disease. However, future studies are required to elucidate the function of fish HKL EVs.

## Conclusion

Very little is known about the biology of fish EVs, their molecular profile or their function. Our previous work identified changes in miRNA expression in Atlantic salmon HKLs as they differentiated during *in vitro* culture (47). Several of the miRNAs identified as being upregulated in Day 5 HKLs (e.g. ssa-miR-146a, ssa-miR-155 and ssa-miR-731) (47) were also identified as upregulated within the EVs derived from Day 5 HKLs by RNA-seq. Then again, the abundance of some miRNAs in EVs were very different to the abundance of miRNAs in HKLs. Together, this indicates that profiling a selection of these miRNAs could both confirm that they originate from HKL EVs (e.g. ssa-miR-125b) and provide useful information about HKL maturation (e.g. expression of ssa-miR-146a, ssa-miR-155 and ssa-miR-731). Used in such a manner, these miRNAs may be useful biomarkers of fish macrophages. Many of the identified miRNAs are also involved in macrophage differentiation and function in both mammals and fish, including ssa-miR-146a and ssa-miR-21a, further suggesting that these miRNAs are involved in immune response and/or macrophage activation. Future studies should focus on functional studies required to test this hypothesis. Thus, our study provides a suitable foundation for future studies on the ability of EVs to serve as indicators of fish immune cell differentiation, activity and their response to stress or disease.

## Data Availability Statement

The datasets presented in this study can be found in online repositories. The names of the repository/repositories and accession number(s) can be found below: https://www.ncbi.nlm.nih.gov/geo/query/acc.cgi?acc=GSE143360.

## Ethics Statement

The animal study was reviewed and approved by Memorial University of Newfoundland’s Institutional Animal Care Committee (18-01-MR; 14-02-MR).

## Author Contributions

Conceptualization: NS, DA, SLC, and MR. Methodology: NS, SLC, and MR. Software: NS, SC, GW, RA, and NW. Validation: NS. Formal analysis: NS, GW, SC, JL, NC, NW, and RA. Investigation: NS. Resources: SLC, MR, and SL. Data curation: NS, GW, SC, SL, NW, and RA. Writing—original draft preparation: NS. Writing—review and editing: NS, GW, SC, NW, JL, NC, DA, RA, SL, MR, and SLC. Visualization: NS. Supervision: MR and SLC. Project administration: NS, SLC, and MR. Funding acquisition, MR and SLC. All authors contributed to the article and approved the submitted version.

## Funding

This study was funded by a Memorial University of Newfoundland Seed grant to SLC (212779), a Natural Sciences and Engineering Research Council of Canada (NSERC) Discovery Grant to MR (341304-2012), a NSERC Discovery Grant to SLC (2017-04630), a Norwegian Research Council grant to RA (280839/E40), and a NSERC PGS D fellowship to NS.

## Conflict of Interest

The authors declare that the research was conducted in the absence of any commercial or financial relationships that could be construed as a potential conflict of interest.
